# Surface deep profile synchrotron studies of mechanically modified top-down silicon nanowires array using ultrasoft X-ray absorption near edge structure spectroscopy

**DOI:** 10.1038/s41598-019-44555-y

**Published:** 2019-05-30

**Authors:** S. Yu. Turishchev, E. V. Parinova, A. K. Pisliaruk, D. A. Koyuda, D. Yermukhamed, T. Ming, R. Ovsyannikov, D. Smirnov, A. Makarova, V. Sivakov

**Affiliations:** 1 Voronezh State University, Voronezh, 394018 Russia; 2 al-Farabi Kazakh National University, Almaty, 050040 Kazakhstan; 3 Leibniz Institute of Photonic Technology, Jena, 07745 Germany; 4 Helmholtz Zentrum Berlin, Berlin, 12489 Germany; 5 Dresden University of Technology, Dresden, 01062 Germany

**Keywords:** Nanowires, Nanowires

## Abstract

Atomic, electronic structure and composition of top-down metal-assisted wet-chemically etched silicon nanowires were studied by synchrotron radiation based X-ray absorption near edge structure technique. Local surrounding of the silicon and oxygen atoms in silicon nanowires array was studied on as-prepared nanostructured surfaces (atop part of nanowires) and their bulk part after, first time applied, *in-situ* mechanical removal atop part of the formed silicon nanowires. Silicon suboxides together with disturbed silicon dioxide were found in the composition of the formed arrays that affects the electronic structure of silicon nanowires. The results obtained by us convincingly testify to the homogeneity of the phase composition of the side walls of silicon nanowires and the electronic structure in the entire length of the nanowire. The controlled formation of the silicon nanowires array may lead to smart engineering of its atomic and electronic structure that influences the exploiting strategy of metal-assisted wet-chemically etched silicon nanowires as universal matrices for different applications.

## Introduction

The unique physico-chemical properties, provided by metal-assisted wet-chemically etched (MAWCE) silicon nanostructures have been attracted considerable attention over the last years^1–3^. The silicon based approaches are certainly favored due to the material abundance and non-toxicity at a high level of materials control and understanding together with a huge industrial infrastructure to account for low production/processing costs and high production yields. For that reason, porous silicon nanostructures (nanowires, nanoparticles) have been gained an enormous interest and employed as the semiconductor material toward hydrogen fuel production such as photoelectrochemical water splitting^4,5^. However, the indirect band gap character of bulk silicon (1.12 eV) and high valence band maximum make impossible the oxidation of water molecule and formation of molecular oxygen. Due to the low band gap energy, the photo- generated electrons and holes are recombined much faster in comparison to wide band gap semiconductors. However, the band gap of nanostructured silicon can be increased by the shrinking of silicon dimension^6,7^. In terms of direct photocatalysis, Si nanowires have extensively demonstrated their use in dye degradation^8^ and palladium-catalyzed organic reactions including Heck coupling, hydrogenolysis, hydrosilylation, and C-H bond functionalization^9^. Theoretical simulations have also indicated that Si nanowires should be a promising photocatalyst for direct water splitting^10^. Since last few years a growing interest on the formation and application of porous silicon nanostructures as photocatalyst can be recognized from the literature overview. Different porous silicon nanostructures have been synthesized and shown significantly enhanced solar-driven hydrogen evolution^11–15^, but the long term stability due to the silicon oxidation and main light absorption in UV spectral region was mentioned as significant disadvantages of silicon photocatalyst. Obviously, SiNWs surface could play an important role for semiconductor possible applications in photocatalysis. In spite of many research works, only weak study efforts have been paid for investigations of MAWCE silicon nanowires surface analysis, especially such fundamental properties as atomic and electronic structure. Any experimental data on the atomic, electronic structure and phase composition peculiarities will be a valuable asset to the general picture of SiNW system physical properties, their technology and applications development. Thus, investigation of the atomic and electronic structure of SiNWs is a crucial scientific task. For precise investigation of the SiNWs formation and interaction between Si and O atoms the use of non-destructive methods sensitive to the phase composition and surface structure is needed. As is well-known, the synchrotron radiation X-ray absorption near edge structure (XANES) technique provides information about the local partial density of free electronic states near the conduction band bottom^16–19^. This technique is sensitive to the local atomic surrounding (for the specific kind of atom – Si or O in our case) in surface nanolayers^16–23^. In comparison to known porous silicon or bottom-up SiNWs structures investigations^18,19,22^ MAWCE SiNWs XANES studies has not been widely presented. One of the great exceptions is ref.^23^, where the origin of light emission in MAWCE SiNWs was investigated, but the origin of surface structure atop of SiNWs in comparison with SiNWs matrices bulk have not been considered, due to analyzed depth value limitations for silicon or oxygen XANES studies in the ultrasoft X-ray region^20^. The role and influence of certain formation parameters can be also influence the surface structure of nanostructured silicon. The main purpose of this work is to establish the electronic structure and peculiarities of the composition of the MAWCE SiNWs array obtained in two different matrix regions, not only from the upper part of the formed SiNWs array, but also from its deeper bulk part after mechanical removal *in situ* of the upper part of SiNWs in an inert atmosphere. The reported approach allows to overcome structure disordering problem, existed for ion-beam etching aimed at depth profiling, that is usually used for XPS studies, but not suitable for local atomic structure sensitive XANES.

## Results and Discussion

**Figure 1:**
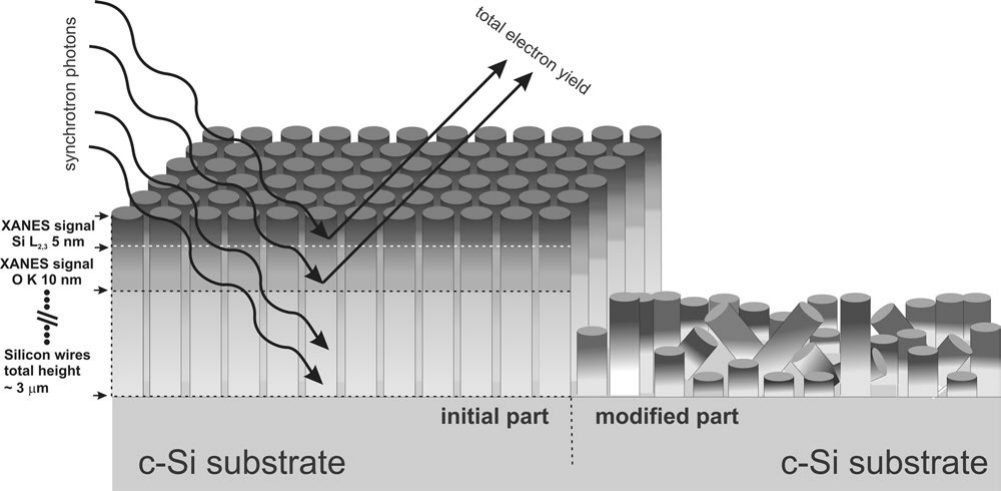
Schematic representation of mechanical surface modification of SiNWs array and analysis depths distribution for discussed absorption edges of synchrotron experiments.

In the present work, to achieve deeper (inside-bulk) part of the formed SiNWs array by surface sensitive XANES technique, each sample was fixed at the sample-plate and half of each sample was mechanically removed in the glove-box under Ar atmosphere. The non-destructed part of each sample is pointed as “initial” and mechanically treated surface (scratched) part is pointed as “modified”. The schematic representation of the sample cross-section before and after mechanical treatment with X-ray spectroscopy information depth is given in Fig. 1.

Silicon nanowires grey color brightness is corresponding for 5 to 10 nm informative depth with respect to the measured silicon L_2,3_ or oxygen K one X-ray absorption edges. After mechanical treatment prepared SiNWs samples were immediately placed inside the Russian-German beamline multi-chamber system to avoid the surface contact with atmosphere. One should be mentioned, that the mechanical surface modification was performed at ambient conditions without any additional heating or presence of active or aggressive environment. This should be realized, just to keep mechanically modified surface parts in their natural physico-chemical state, in inert atmosphere prior to transferring into UHV spectrometer chambers. Moreover, we have to underline the necessity of electron microscopy control after surface treatment to estimate the possible transition area between initial and modified surfaces, just to avoid certain mismatch of the synchrotron light beam position. Finally, the suggested approach seems to be quite promising for analyzing of deeper parts of structures having surface that is noticeably developed not only in plane, but through their whole thickness up to bulky part (e.g. porous layers, whiskers or nanowires array, etc).

**Figure 2:**
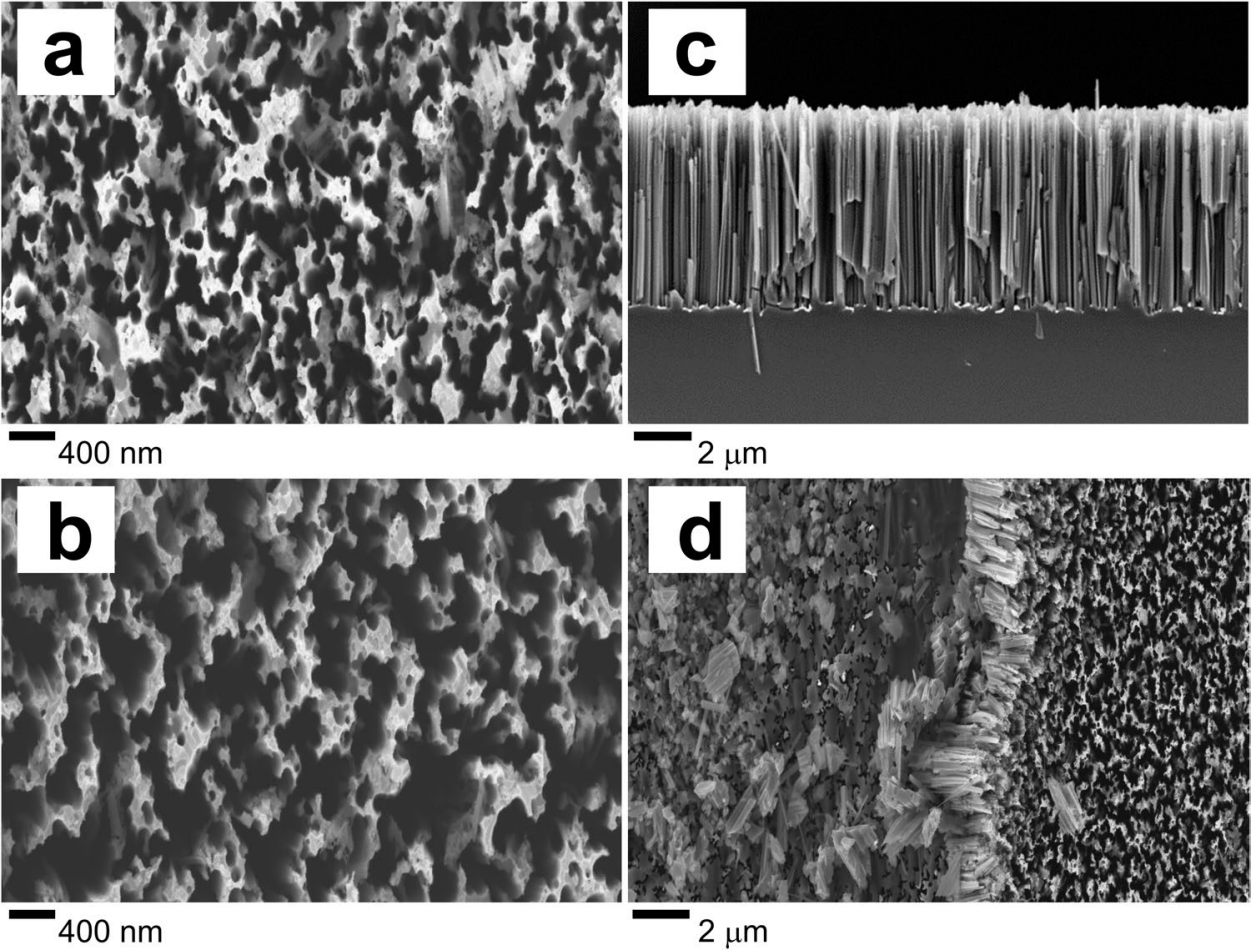
SEM micrographs of: (**a**) SiNW-15 nanostructured silicon surface using 15 s silver deposition in the first MAWCE etching step; (**b**) SiNW-45 nanostructured silicon surface using 45 s silver deposition in the first MAWCE etching step; (**c**) typical cross sectional view for MAWCE SiNWs array; (**d**) *in-situ* mechanically modified nanostructured silicon surface.

The scanning electron microscopy (SEM) studies of the formed SiNW arrays morphology are presented in Fig. 2. Figure 2a,b shows planar views of silicon nanowires prepared at different silver deposition times in the first MAWCE process step and Fig. 2c shows typical SEM cross sectional view of SiNWs. The silver deposition time in the first etching step strongly influences the density and/or distance between neighboring silicon nanostructures, that influence the final morphology of the nanostructured silicon matrix and plays the role as a native mask. As well as the pre-nature of the starting wafers, like doping level of silicon substrate, strongly influence the morphology of SiNWs in MAWCE process as was published by Geyer *et al*.^24^. Higher density of nanowires due to the formation of smaller silver nanoparticles was observed in the sample with 15 s silver deposition time as shown in Fig. 2a.

In comparison to SiNW-15, the sample obtained at a longer deposition time of silver (SiNW-45), provides a greater distance between adjacent nanowires (see Fig. 2a,b). That means that by varying the silver deposition time, different surface porosity of the SiNWs array can be achieved. The *in-situ* mechanically modified nanostructured silicon surface is presented in Fig. 2d. The border between mechanically treated and non-treated SiNWs part is clearly visible (XANES studies on these parts will be discussed below). As can be seen from the combination of Figs 1 and 2, the mechanically altered surface area contained a wire torn at approximately the same depth (~ 2 µm), or their fragments lying above the surface at different angles in relation to the incident beam. Thus, the bulk part (deeper SiNWs sidewalls) of the wires is available for measurement with XANES Si L_2,3_ and O K spectra.

**Figure 3:**
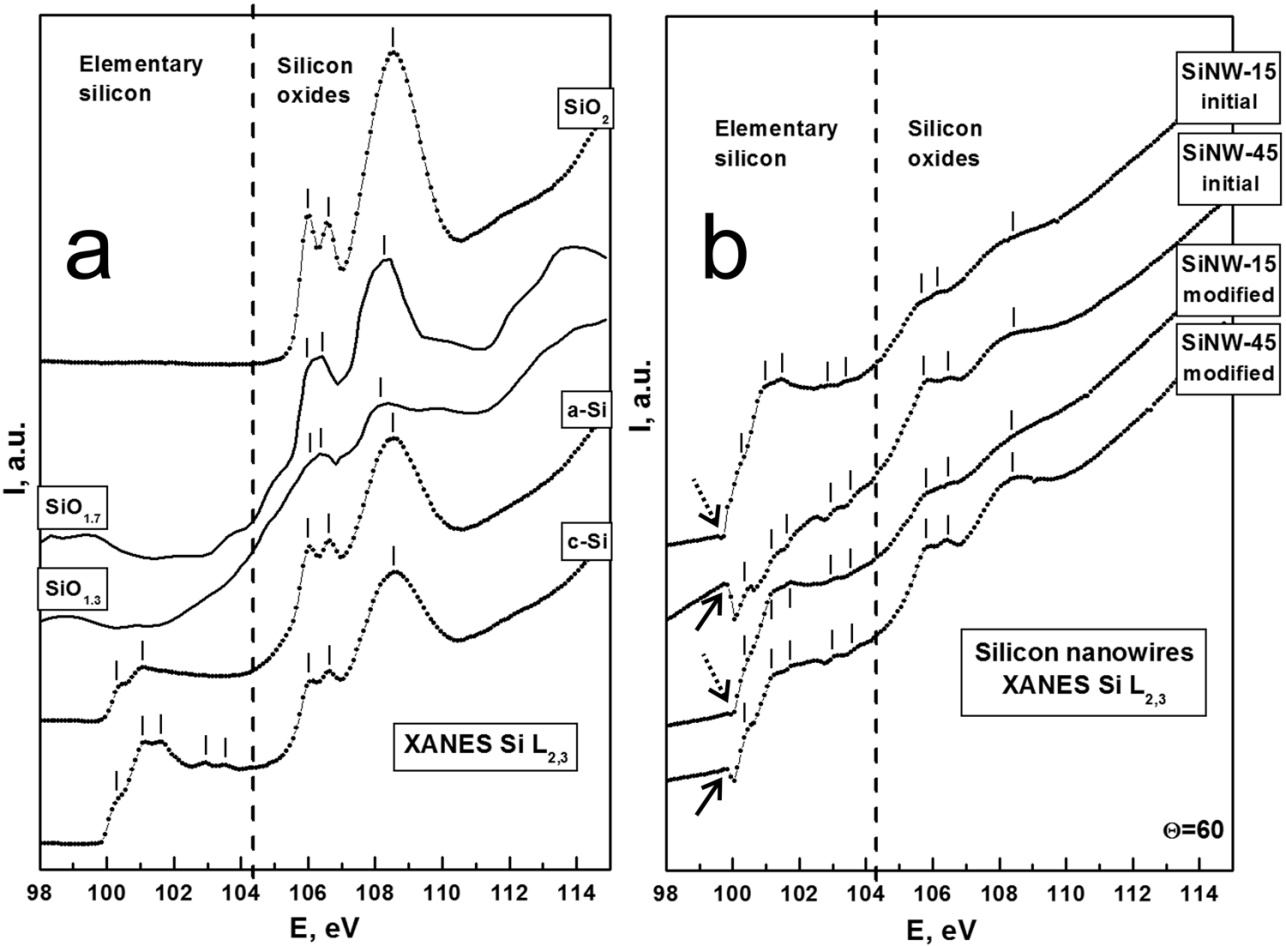
(**a**) XANES Si L_2,3_ spectra for the references (from down to top) crystalline silicon c-Si, amorphous silicon a-Si, silicon suboxides SiO_1.3_ and SiO_1.7_ taken from ref.^21^, thermally grown 40 nm film of silicon dioxide SiO_2_; (**b**) XANES Si L_2,3_ registered from the initial arrays obtained under different etching time (15 and 45 sec) and their *in-situ* mechanically modified surface parts. Arrows indicate the presence of differently pronounced dip.

XANES spectra were registered relative to L_2,3_ absorption edge of silicon for different references as presented in the Fig. 3a. The separation between elementary silicon part (photons energies below 104 eV) and silicon oxides part (photons energies higher than 104 eV), accompanied with well-expressed fine structure, allows easily consider changes in local atomic surrounding over the analyzed layer.

The observed fine structure close to the main absorption edge (~100 eV) is presented due to the spin-orbital splitting of silicon L_2,3_ core level, that leads to formation of double peak features at photons energies ~101–102 eV and 102–104 eV in the crystalline silicon wafer (c-Si) reference spectrum. This developed density of state distribution fine structure is specific to the ordered character of silicon atoms grid in the analyzed surface layer^25,26^. At the same time, for disordered system (few µm a-Si reference thick layer on the c-Si wafer, Si L_2,3_ XANES in Fig. 3a) the dome-like blurred distribution of the density of states is observed in the 100–104 eV photon energy range^19,25,27^.

The absorption peaks at the photon energies higher than 104.2 eV are corresponding for the silicon oxide part. Single crystalline silicon (c-Si) and amorphous silicon (a-Si) references were covered by natural silicon oxide with spectral fine structure similar to the SiO_2_ reference (10 nm thermally grown SiO_2_ film on c-Si wafer). Additionally, to the Fig. 3a, we insert two of silicon sub-oxides films spectra taken from ref.^21^. It seems to be, that for oxidation degree lower than two density of states distribution is higher comparing to stoichiometric SiO_2_ causing additional electronic states appearance upper 2 eV from conduction band bottom (~104–106 eV).

Si L_2,3_ XANES spectra of the “initial” and “modified” SiNW arrays are presented at in Fig. 3b. The spectrum obtained for the “initial” SiNW-15 array is more pronounced fine structure in the elementary silicon absorption edge that corresponds to ordering in Si atoms in the formed silicon nanowires. From another side, for the initial SiNW-45 spectrum distorted pointing out the more etched wires with still ordering of Si atoms is presented. In all spectra of nanowires arrays the dip of X-ray photons absorption at ~100 eV is presented that most likely caused by falling synchrotron radiation beam interaction with wires array. This dip is much more noticeable for SiNWs-45 nanowire arrays, but can be also observed for SiNW-15 (solid and dashed arrows in Fig. 3b, respectively). Previously, such effects were also observed, where due to the differently oriented Si nanoparticles or nanostructures to the falling synchrotron X-ray photons the strong decrease of absorption was detected near the main Si L_2,3_ absorption edge^27,28^. The detailed and specific synchrotron studies of the nature of this dip disposed near the L_2,3_ silicon core level resonance are planned as the further scientific direction considering silicon nanowires arrays specific properties.

The low silicon oxidation degree should be underlined by weakly pronounced silicon oxide fine structure (hν > 104.2 eV) for all registered SiNWs spectra. Nevertheless, for SiNW-45 array the fine structure in silicon oxide region is better expressed. The relative spectral intensity rise at the energy ranges of 104–106 eV (Fig. 3b) can be the evidence of the presence of silicon suboxides^21^ over the developed SiNWs surface. The same behavior we observed in our previous XANES studies of electrochemically produced porous silicon obtained under different etching time and stored in ambient conditions (ageing effect) for different and quite prolonged for natural oxidation time^19,29^. Finally, it should be noted that in general Si L_2,3_ XANES spectra features positions and their relative intensities are quite the same for “initial” and “modified” surfaces of the SiNW arrays. This fact means the homogeneous NWs surface composition and electronic structure for all their length (top and down).

**Figure 4:**
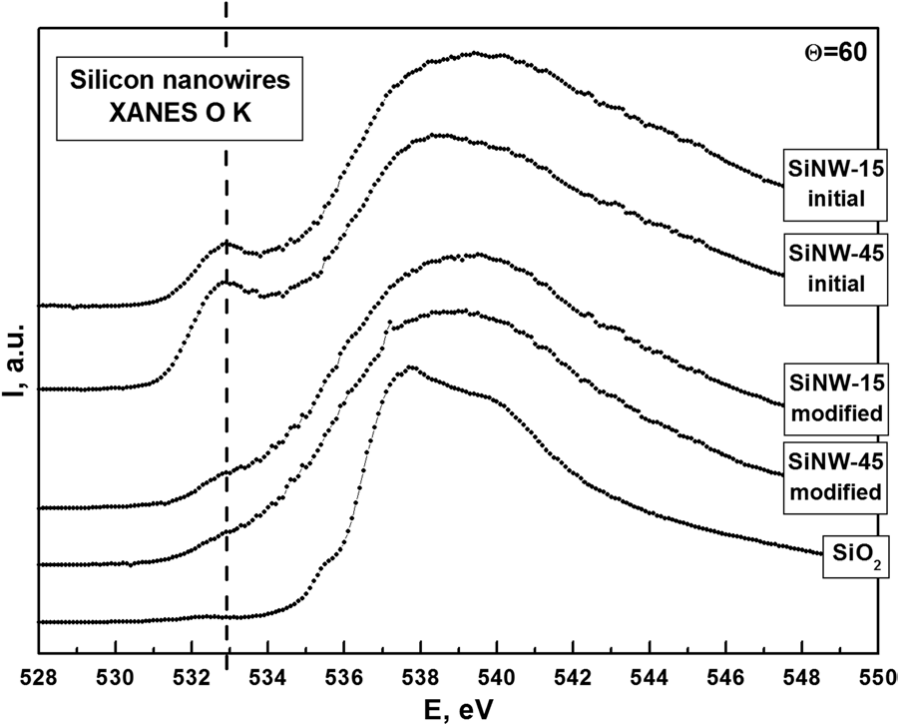
XANES O K spectra for SiNW arrays in comparison with thermally grown SiO_2_ reference film.

Figure 4 shows the O K XANES spectra for the investigated SiNW arrays and comparison with the XANES O K spectrum for the 40 nm thermally grown SiO_2_ reference film. The lowest energy feature at ~533 eV originates from the silicon oxide irregularities compared with the stoichiometric SiO_2_^30,31^. This feature is more pronounced for both “initial” SiNW arrays (15 and 45), e.g. observed as the well distinguished peak at 533 eV. These observations are in a good agreement with the comparative analysis of Si L_2,3_ XANES fine structure, performed above in the silicon oxide range (photons energies higher than 104 eV) by the local surrounding of oxygen atoms specificity.

In summary, we can conclude that the top part of the formed SiNWs array is more textured (structured) with the highest probability of different deviations from stoichiometric SiO_2_ (e.g. dense Si-O tetrahedra packaging for thermally grown SiO_2_ reference film) including presence of suboxides confirmed by consideration of XANES Si L_2,3_ spectra (as shown in Fig. 3). On the other hand, the down (deeper) region of SiNWs is not highly textured (i.e. structured) and deviation from stoichiometric SiO_2_ is less probable. As evidence for that statement, the suppression of signal at 533 eV (instead of the distinguished feature) for the “modified” samples parts is clearly visible in Fig. 4. The rest dome-like main feature of XANES O K spectra taken from SiNWs (hν > 534 eV) is compared with the fine structures of SiO_2_ reference, that confirms the loose nature of the natural oxide similarly covering formed SiNW arrays.

Finally, we should mention that synchrotron studies can be performed near silicon K core level resonance. The probing depth for Si K edge XANES is established around 65 nm^20^ that is deeper than known 5 nm for Si L_2,3_ XANES. There are known a couple of papers, including ones, subjected by Si nanowire arrays studies^20,30,32,33^ based on XANES K-edge spectroscopy results. On the other hand, the 65 nm probing depth is sufficiently smaller than removed Si wires arrays parts with height that is more than few micrometers (as shown in Figs 1 or 2). This makes studies of Si K edge XANES useful, but not so informative in comparison to the presented results.

## Conclusions

The simple approach of highly surface sensitive technique application for synchrotron characterization of structures with highly developed surface morphology has been proposed for the first time. SiNWs surface before and after mechanical altering (initial and modified) were investigated by the high energy resolution synchrotron XANES technique. Local surrounding of the silicon and oxygen atoms character confirms the uniformity of the silicon oxides coverage of formed SiNW arrays at the surface atop layers, that did not exceed 10 nm, and their bulk part after *in-situ* mechanical removal (approx. 2 µm) of the formed nanowires. The results obtained by us convincingly testify to the homogeneity of the phase composition of the side walls of SiNWs and the electronic structure in the entire length of the nanowire. Silicon atoms ordering and suboxides presence together with formed SiNWs density plays an important role in the electronic structure and composition of silicon nanowires arrays. The engineering of atomic and electronic structure of nanostructured silicon is a main factor for the exploiting SiNW arrays as universal matrices for different application fields and can be precisely determined by X-ray spectroscopy.

## Methods

The array of silicon nanowires was fabricated by MAWCE approach, in details previously described in our earlier publications^6^. The concentration of silver (Ag) ions in the first etching step strongly influences the density and/or distance between neighboring silicon nanostructures, which finally can influence the material penetration to the porous silicon matrix by the further matrix functionalization^34^. The nanostructured silicon surface formation involves two main steps: (1) silver nanoparticles deposition on the silicon wafer surface using aqueous solutions of 0.01 M silver nitrate and 5 M hydrofluoric acid (HF) in the volume ratio 1:1 (v/v) for several seconds; (2) the subsequent anisotropic wafer etching occurs in the HF/H_2_O_2_ solution. Silicon wafers covered with Ag nanoparticles are immersed in a 5 M HF and 30% H_2_O_2_ in the volume ratio 10:1 (v/v) etching solution for several min at ambient condition. Afterwards, the arrays were rinsed several times in de-ionized water and dried at room temperature. The nanostructured arrays were treated in a concentrated 65% nitric acid for 10 min to remove Ag nanoparticles from the SiNWs sidewalls. SiNW-15 and SiNW-45 samples were produced at 15 s and 45 s silver deposition time in first etching step, respectively. The morphology analysis of nanostructured silicon surfaces was carried out by Carl Zeiss ULTRA 55 scanning electron microscope (SEM).

High resolution XANES spectra relative to Si L_2,3_ and O K core levels were obtained at the Russian-German Lab end-station of the Berlin synchrotron radiation storage ring BESSY II (Helmholtz Zentrum Berlin)^35^. Energy resolution was about 0.05 eV with beam current in the ring of ~250 mA operating in top-up mode with the beam diameter, on the studied sample surface, less than 10^−2^ m. Samples were located at the standard “Omicron” type sample holder with the 60° synchrotron light grazing angle Θ relative to the sample surfaces as is indicated in the Figs 3 and 4. Probing depth was considered as less than 5 nm for Si L_2,3_ according to ref.^20^ and less than 10 nm for O K edges according to ref.^36^. Vacuum in the analytical and preparation chambers was continuously kept at 10^−9^ Torr during survey of spectra. Total electron yield regime was used in sample (drain) current detection mode. Signal of XPS controlled pure Au foil (I_0_) was used for standard procedures of the measured spectra normalization^16^.

## Data Availability

The data that supports the findings of this study are available from the corresponding author on reasonable request.
